# The Double-Edged Nature of Patient-to-Patient Interactions in Digital Healthcare Communities: A Qualitative Study of Patients with Type 2 Diabetes in China

**DOI:** 10.3390/healthcare14183025

**Published:** 2026-09-15

**Authors:** Tang Yao, Jiayi Liu, Yuhang Zhang, Jingyi Cao, Huarui Cao

**Affiliations:** 1 School of Economics and Management, Beihang University, Beijing 100191, China; yaot@buaa.edu.cn (T.Y.);; 2 MOE Key Laboratory of Complex System Analysis and Management Decision, Beihang University, Beijing 100191, China; 3 School of Management, Tianjin Normal University, Tianjin 300387, China

**Keywords:** digital healthcare community, patient-to-patient interaction, future self-continuity, quality of life, sequential qualitative study

## Abstract

**Background/Objectives**: Digital healthcare communities enable patients to exchange emotional support, experiential knowledge, and social companionship but may also expose patients to distress, misinformation, conflict, and relational harm. This study identified beneficial and harmful patient-to-patient interaction practices and explored how patients interpreted these experiences in relation to future self-continuity and perceived quality of life. **Methods**: A two-phase sequential qualitative design was used. Phase 1 analyzed 871 valid discussion topics from a Chinese digital diabetes community. Phase 2 involved two rounds of semi-structured interviews, conducted approximately two months apart, with 12 adults with type 2 diabetes purposively sampled across six interaction categories identified in Phase 1. Interview data were analyzed using a hybrid deductive–inductive thematic approach with longitudinal within-case and cross-case comparison. **Results**: Phase 1 identified 25 patient-to-patient interaction practices encompassing both beneficial and harmful practices across emotional, informational, and social domains. In Phase 2, participants described supportive interactions in terms of greater hope, perceived control, and social belonging, whereas harmful interactions were described in terms of fear, uncertainty, self-doubt, and mistrust. Participants further described how these experiences related to their future selves. Participants who experienced their future selves as more attainable, recognizable, and personally meaningful also described greater hope, emotional stability, meaning, and engagement in self-management. Participants also perceived connections between these future-oriented interpretations and psychological, existential, and physical aspects of their quality of life. However, these findings should not be interpreted as evidence of a causal relationship between future self-continuity and quality of life. **Conclusions**: Patient-to-patient interactions in digital healthcare communities may be experienced as both supportive and harmful and may be related to how patients interpret the relationship between their present and future selves. Future self-continuity provides an interpretive perspective for understanding how online peer experiences may be associated with patients’ perceptions of well-being within the observed time frame. These findings should be interpreted within the specific sociocultural and healthcare context of a Chinese digital diabetes community and warrant further investigation across different populations and digital healthcare settings.

## 1. Introduction

Digital healthcare communities have become important spaces in which patients discuss illness, exchange experiential knowledge, and develop relationships with others facing similar health challenges [1]. In this context, patient-to-patient interaction refers to the reciprocal exchange of illness-related information, practical experience, emotional responses, and social companionship between patients [2]. Patients are therefore not merely recipients of online information or support. The same patient may seek reassurance and guidance in one interaction and provide encouragement, knowledge, or companionship in another. These exchanges have been associated with feeling understood, reduced isolation, and greater knowledge and confidence, but also with emotional distress, inaccurate advice, interpersonal conflict, privacy violations, and commercially motivated misinformation [1,2,3]. Recent developments in digital health and rehabilitation technologies further highlight the growing role of technology-enabled healthcare in supporting patient needs and being relevant to public health outcomes [4]. Although interaction may contribute to either value co-creation or value co-destruction [5], research on digital healthcare communities has predominantly framed patient exchanges as sources of social support, empowerment, and beneficial health outcomes. Harmful exchanges have more often been addressed as isolated concerns, such as misinformation or privacy risk, rather than examined alongside beneficial exchanges as part of an integrated interaction system. Consequently, the specific practices through which patients experience beneficial or harmful interactions remain insufficiently classified. The first research question was therefore “What beneficial and harmful patient-to-patient interaction practices occur in digital healthcare communities?”

Beyond their immediate emotional and informational consequences, patient-to-patient interactions may also be related to how patients understand themselves across time. Existing research has primarily examined patient-to-patient interactions through the lens of social support, empowerment, and information exchange [1,2]. However, these perspectives mainly explain patients’ immediate experiences. For individuals living with chronic illness, a fundamental psychological challenge lies in maintaining a coherent connection between their current identity and anticipated future self. Therefore, future self-continuity provides a temporal interpretive lens for examining how patients relate patient-to-patient interactions to their longer-term adaptation [6]. Future self-continuity refers to the perceived connection between one’s present self and the person one expects to become, including the extent to which these temporal selves are experienced as parts of the same continuing identity [6,7]. This perception is particularly relevant for patients living with chronic illness. Diagnosis, continuing treatment, uncertain disease progression, and bodily changes may be associated with disruptions to established life plans and with reconsideration of patients’ identities, capabilities, relationships, and future possibilities [8]. Within digital healthcare communities, other patients’ stories, emotional expressions, practical advice, and descriptions of illness trajectories may therefore serve as interpretive resources through which patients imagine what their own future lives could become. Supportive accounts may be associated with perceptions of the future as manageable and connected to present action, whereas distressing or misleading accounts may be associated with perceptions of the future as threatening, uncertain, or detached from the present self. Nevertheless, research on digital healthcare communities has focused mainly on support, empowerment, and disease management [9], while research on future self-continuity has largely examined intertemporal decision making and individual health behavior [6,7]. Little is known about how patients interpret particular interaction experiences and whether these experiences are perceived as relevant to the relationship they perceive between their present and future selves. The second research question was therefore “How do patients interpret beneficial and harmful patient-to-patient interaction experiences, and how, if at all, are these experiences perceived as relevant to the relationship between their present and future selves?”

Perceived changes in future self-continuity may also be related to patients’ quality of life. Quality of life is a broad and multidimensional construct that reflects individuals’ evaluations of their physical condition, psychological well-being, and life circumstances. In healthcare research, it is typically understood as a subjective and experiential indicator of how illness and its management affect everyday life [10,11]. Rather than attempting to capture all possible domains, this study adopts a focused health-related quality-of-life perspective. Specifically, we draw on the McGill quality-of-life framework and focus on physical, psychological, and existential domains. The physical domain reflects symptom-related experiences, the psychological domain captures emotional well-being, and the existential domain concerns individuals’ sense of meaning and purpose, how they perceive themselves, and the extent to which they feel a sense of control over their lives [12]. These domains were selected because they are both theoretically grounded and particularly salient in participants’ narratives regarding chronic illness management and everyday well-being. Importantly, quality of life is conceptually distinct from future self-continuity. While future self-continuity refers to the perceived connection between present and future selves, quality of life reflects evaluations of current well-being [7,12]. Although the existential dimension involves meaning, self-perception, and perceived control, it refers to how individuals evaluate their current lives and sense of self, whereas future self-continuity concerns the perceived connection between one’s present and future selves across time. To maintain conceptual clarity, we distinguish between references to connections between present and future selves and evaluations of current physical, psychological, and existential conditions. This temporal distinction is nevertheless relevant to interpreting participants’ accounts because perceptions of the future may shape how patients make sense of present difficulties and the value of current efforts. In participants’ narratives, perceived continuity with a viable future self was sometimes accompanied by accounts of greater hope, meaning, perceived control, or willingness to engage in health-protective behaviors [7,13,14]. Conversely, perceived disruption of this connection was sometimes accompanied by fear, helplessness, or a reduced sense that present self-management was worthwhile. Previous research has demonstrated a direct association between online social support and patients’ quality of life [9]. However, this approach does not fully explain how patients interpret the relationship among interaction experiences, their future selves, and different dimensions of quality of life over time. The third research question was therefore “How do patients describe the relationship between their experiences of patient-to-patient interactions, perceived changes in future self-continuity, and subsequent psychological, existential, and physical quality of life?”

To address these questions, this study adopted a two-phase sequential qualitative design. In the first phase, qualitative content analysis was conducted on 871 valid discussion topics obtained from the Sweet Home digital healthcare community, which serves patients with type 2 diabetes. This phase identified the specific beneficial and harmful practices through which diabetes patients interacted with one another and provided the empirical foundation for the subsequent interviews. In the second phase, 12 diabetes patients participated in two rounds of semi-structured in-depth interviews. The first round examined how patients interpreted particular online interaction experiences and how they related these experiences to their understanding of the relationship between their present and future selves. Two months later, a second round of interviews explored how participants described perceived changes in future self-continuity in relation to their subsequent psychological, existential, and physical quality of life. Consistent with the exploratory nature of qualitative inquiry, the study prioritized depth and information richness rather than statistical generalizability. This sequential design connected the identification of observable interaction practices with patients’ interpretations of those practices and their later accounts of quality of life, thereby enabling the three research questions to be examined as related stages of an interpretive and temporal process.

This study makes three contributions. First, it develops a practice-level classification that examines beneficial and harmful patient-to-patient interactions within the same emotional, informational, and social domains. This approach moves beyond treating online peer exchange as uniformly supportive or examining misinformation, conflict, and privacy risks as unrelated problems. Second, the study introduces future self-continuity as an interpretive lens for understanding how patients connect immediate peer experiences with anticipated identities, health trajectories, and future possibilities. It thereby extends future self-continuity from an individual temporal judgment to a perception that may be understood in relation to relational and contextual experiences. Third, the sequential qualitative design links naturally occurring online practices with patients’ interpretations across two interview points, showing how participants related interaction experiences to their subsequent accounts of psychological, existential, and physical quality of life. Together, these contributions provide a more differentiated and patient-centered account of how digital healthcare communities may be associated with supportive or harmful experiences relevant to longer-term well-being.

## 2. Materials and Methods

This study was designed and reported with reference to the Consolidated Criteria for Reporting Qualitative Research (COREQ) and the Standards for Reporting Qualitative Research (SRQR) [15,16]. A two-phase sequential qualitative approach was used to examine online patient-to-patient interaction at three connected levels: the forms that these interactions took in a digital healthcare community, patients’ interpretations of the relationship between particular interaction experiences and their perceptions of their future selves, and the ways in which these future-oriented interpretations were subsequently described in relation to quality of life. The following sections describe the study design, online community data, participant recruitment and sampling, data collection, data analysis, ethical considerations, and strategies used to enhance trustworthiness.

### 2.1. Study Design

A two-phase sequential qualitative design was employed. This design was selected because the study sought first to identify and classify online patient-to-patient interaction practices and then to develop an in-depth understanding of how patients interpreted these experiences over time. Qualitative inquiry was appropriate because the research questions focused on the meanings patients assigned to their experiences rather than the statistical testing of predetermined relationships [17].

Because this study is a sequential qualitative inquiry rather than an algorithmic, predictive, or computational-method study, it does not involve comparisons with state-of-the-art computational methods or the use of model-performance metrics. The purpose of the study was to identify and interpret patient-to-patient interaction practices and participants’ perceived implications of these experiences over time, rather than to develop, optimize, or benchmark the performance of a predictive model or computational method. Accordingly, percentage or absolute performance improvements over existing methods are not applicable to the present study. Instead, methodological rigor was ensured through systematic qualitative procedures, including independent coding, iterative refinement of the coding framework, inter-coder agreement assessment, consensus discussions, and maintenance of an audit trail.

The first phase involved an inductively oriented qualitative content analysis of naturally occurring secondary textual data from a digital healthcare community. The analysis was primarily data-driven and did not begin with a predetermined coding framework. Previous literature on healthcare, online social support, and patient interaction served as a sensitizing resource rather than as a source of predefined coding categories [18,19]. This phase aimed to identify the specific practices that reflected beneficial or harmful forms of patient-to-patient interaction and to develop a category structure grounded primarily in the empirical material. The findings established the empirical basis for examining patients’ experiences in greater depth during the second phase.

The second phase adopted a longitudinal qualitative approach and consisted of two rounds of semi-structured in-depth interviews with the same participants. The first-round interviews explored how patients interpreted particular beneficial and harmful interaction experiences and how they described these experiences in relation to their perceptions of their present and future selves. Two months later, the second-round interviews explored how participants described their future-oriented self-perceptions and whether these accounts were reflected in their subsequent descriptions of psychological, existential, and physical quality of life. Repeated qualitative interviews enable researchers to examine how experiences and meanings develop across time [20,21] rather than relying solely on a single retrospective account.

The two phases were connected sequentially. The first phase addressed what beneficial and harmful interaction practices occurred in the digital healthcare community, while the second phase examined how patients made sense of these practices and described their implications over time. The design therefore progressed from identifying interaction practices to examining participants’ interpretations of perceived changes in future self-continuity and their subsequent accounts of quality of life.

### 2.2. Phase 1: Online Community Data and Content Analysis

Given the premise that patient-to-patient interaction encompasses both beneficial and harmful aspects, our primary focus is on elucidating the following question: What are the specific forms and characteristics of beneficial and harmful patient-to-patient interaction in the digital healthcare context? To address this question, we investigated a typical healthcare forum and analyzed its content to identify the themes underlying the dual nature of patient-to-patient interaction.

#### 2.2.1. Research Setting

Phase 1 focused on type 2 diabetes, a prevalent chronic condition requiring sustained management. This setting was selected for three reasons. First, diabetes constitutes a substantial public health burden in China. In 2024, the National Health Commission of China reported that approximately 140.9 million people in the country were living with diabetes, with type 2 diabetes accounting for approximately 90% of cases [22]. Second, type 2 diabetes requires patients to engage continuously in illness management both within and beyond formal healthcare settings [23,24]. Digital communities may supplement episodic professional care by providing opportunities for patients to exchange disease-related knowledge, practical experience, emotional support, and companionship [25,26]. Third, the accessibility and relative anonymity of digital environments may also facilitate misleading information, interpersonal hostility, privacy violations, and other harmful activities [27]. This combination of supportive and potentially harmful exchanges made a digital diabetes community an appropriate setting for examining the dual valence of patient-to-patient interaction. Sweet Home, a well-established Chinese digital community serving people living with diabetes, was selected as the research setting. At the time of data collection, the community had more than 220,000 registered members and provided discussion spaces related to treatment, self-management, emotional concerns, and everyday life. The social and relational features of online health communities in the Chinese context further supported the examination of how patients exchanged resources, shared experiences, and engaged with one another in managing chronic illness [9]. Overall, this setting enabled the study to capture naturally occurring online patient-to-patient interactions within the continuing management of chronic illness.

#### 2.2.2. Data Source and Sampling

We collected data from Sweet Home during October 2025. We used Python 3.14 and adopted a systematic interval sampling approach to retrieve online data. At the time of data collection, discussion-topic pages on the platform were ordered in reverse chronological order according to posting time. Consistent with the approach suggested by previous research [9], we used a skip interval of 10 pages and extracted 30 webpages, with approximately 30 discussion topics per page, yielding 907 initially retrieved discussion topics. This sampling procedure was intended to distribute the retrieved discussion topics across different portions of the available online content and reduce potential selection bias [28]. The sampling therefore followed the platform’s ordered discussion archive rather than a predefined calendar interval. For the purposes of this study, a discussion topic comprised the initiating post together with the replies and comments contained in the corresponding discussion thread. We manually reviewed all retrieved topics and excluded 36 invalid entries, such as entries consisting only of symbols or meaningless characters (e.g., “#%@&*^”), leaving 871 valid discussion topics for qualitative analysis. Because the analyzed posts were publicly accessible, the study did not intentionally collect or retain personally identifying information for analysis. Any usernames or potentially identifying details encountered during data retrieval were removed before analysis.

Sweet Home is a Chinese-language digital healthcare community. All Phase 1 online materials were originally posted in Simplified Chinese, and all coding and qualitative content analysis were conducted using the original Chinese-language dataset. Quotations included in the manuscript were translated into English during manuscript preparation only; translation was not part of data collection or the initial coding process. Selected Chinese quotations were reviewed in their original context and translated into English by two researchers, T.Y. and H.C., who are proficient in both Chinese and English. The researchers carefully considered the original wording and contextual meaning when preparing the English translations and cross-checked the translated quotations against the Chinese originals to minimize potential loss or distortion of meaning. Where necessary, minor linguistic adjustments were made to improve readability while preserving the intended meaning of the original statements.

#### 2.2.3. Data Coding

The inductively oriented qualitative content analysis focused on identifying categories of patient-to-patient interaction within the digital healthcare community. The primary analytical task was to identify recurring practices that reflected the beneficial and harmful forms of such interaction. Previous literature [9,23,24] was used as a sensitizing resource to inform the researchers’ attention to potentially relevant aspects of patient-to-patient interaction, rather than as a predetermined coding framework. Three coders, J.L., Y.Z., and J.C., had backgrounds in health-related research and received training in qualitative research. They independently read the complete dataset at least twice and generated initial codes directly from the empirical material.

The coding unit was each valid discussion topic (n = 871). Because each discussion topic included the initiating post and its associated replies and comments, the coders reviewed the complete available thread when interpreting patient-to-patient interaction practices and considered the surrounding conversational context rather than coding isolated posts or comments. Multiple interaction practices could be identified within a single discussion thread when more than one analytically distinct form of interaction was present. However, for the six-category classification reported in Table 1, each discussion topic was assigned to the category representing its predominant interaction practice.

The coding framework was then refined using the constant comparative method through an iterative process of reading, comparing, evaluating, and revising codes until a coherent category structure was developed [29]. The three broad categories of emotional interaction, informational interaction, and social interaction were subsequently developed from the coded data. For the category-level proportions reported in Table 1, the number of valid discussion topics assigned to each category was divided by the total number of valid discussion topics (n = 871) and multiplied by 100. Accordingly, the six category-level proportions were mutually exclusive and summed to 100%, although multiple interaction practices could be identified within an individual thread during the preceding practice-level coding. These proportions were used descriptively to characterize the distribution of interaction categories within the analyzed corpus and were not intended as estimates of the prevalence of these interaction practices among Sweet Home users.

Inter-coder agreement was assessed using Holsti’s coefficient of reliability (CR) [30]. For each pair of coders, the coefficient was calculated as CR = 2M/(N_1_ + N_2_), where M represents the number of coding decisions on which the two coders agreed, and N1 and N2 represent the total number of coding decisions made by the respective coders. For the three coders, Holsti coefficients were calculated for each pair of coders and then averaged to obtain an overall agreement coefficient. The coding unit for this calculation was each valid discussion topic (n = 871). The resulting mean coefficient was 0.87, exceeding the recommended threshold of 0.80 [30]. Disagreements were subsequently resolved through discussion and consensus. These procedures and values are consistent with the original methodological record.

#### 2.2.4. Content Analysis

The qualitative content analysis identified three broad domains of patient-to-patient interaction in the digital healthcare community: emotional interaction, informational interaction, and social interaction. Each domain contained both beneficial and harmful interaction practices.

*Emotional interaction*. The analysis shows that a critical category of online patient-to-patient interaction involves patients’ sense of affection and emotion, consistent with the findings of a broad range of research [9,31]. Patients interact with each other insofar as they want to express or share emotions about their experiences of the processes and outcomes of disease treatment. For example, patients who are satisfied with their service experiences often want to affirm their hopes for the future, while dissatisfied patients vent their negative feelings by expressing disappointment or even posting hostile or disrespectful content, which may be associated with more negative attitudes, reduced hope, and weaker confidence in continuing treatment.

*Informational interaction*. Another category pertains to patients’ inclination to argue about and comment on the complexity and subtleties of user interactions and shared experiences on the virtual platform, or the informational interaction patients may experience in digital settings [32]. For example, by conversing with others without the limitations of time and space on the Internet, patients can adequately exchange their accumulated knowledge of the disease with others, beyond the limited opportunities for information exchange during routine clinical encounters. Some suggestions and information on the forum may be perceived as helpful for patients’ physical and psychological well-being [9,25]; however, some unsupported or potentially misleading suggestions, quarrels, overly subjective complaints, and slander may be associated with negative perceptions of their health and treatment.

*Social interaction*. The final category of patient-to-patient interaction entails patients’ desire for social connections derived from their exchange with others, or social interaction. Scholars have argued that patient value co-creation, including interaction, occurs within broader societal systems. Individuals make sense of reality through activities shaped by social interactions, as well as by their roles and positions within these systems [23]. In the healthcare context, patients wish to bond or simply communicate with other people, especially when they experience insufficient social support or a sense of psychological emptiness associated with the long-term effects of chronic illness. For example, existing research has demonstrated that online companionship may contribute to individuals’ sense of belonging within patient groups [9]. However, some actions with the goal of social interaction may also be associated with harmful experiences, such as when a patient’s privacy is unintentionally or even intentionally disclosed or a person tries to make “friends” to promote untested or even fake medical products or services to others.

In line with the conceptualization of beneficial and harmful patient-to-patient interaction in the digital healthcare setting, we identified (1) six themes of emotional interaction, five themes of informational interaction, and four themes of social interaction within the beneficial interaction category and (2) two themes of emotional interaction, six themes of informational interaction, and two themes of social interaction within the harmful interaction category. We summarize these in Table 1, including the effect, category, definition, activity theme, representative quote, and proportion of discussion topics assigned to each category within the analyzed corpus.

### 2.3. Phase 2: Participants and Sampling

Phase 2 extended the findings of Phase 1 by examining how patients interpreted experiences associated with the six identified categories of beneficial and harmful patient-to-patient interaction. The six categories identified in Phase 1 were used as an organizing framework for purposive sampling, rather than as a predetermined framework for the subsequent thematic analysis. This strategy was intended to ensure representation of contrasting forms of patient-to-patient interaction identified in the online community while allowing participants to describe experiences and meanings beyond these categories. Assignment to a category reflected the focal interaction experience used for sampling and did not imply that participants experienced only one type or valence of patient-to-patient interaction.

The final sample comprised 12 patients, including two participants whose focal interaction experiences were associated with each category: beneficial emotional interaction, beneficial informational interaction, beneficial social interaction, harmful emotional interaction, harmful informational interaction, and harmful social interaction. The same participants were invited to complete both rounds of interviews.

#### 2.3.1. Data Collection

Data were collected between December 2025 and February 2026 through two rounds of semi-structured in-depth interviews conducted approximately two months apart. The interview guide was developed from the six categories of patient-to-patient interaction identified in Phase 1 and was structured around the second and third research questions. Semi-structured interviewing enabled the research team to address consistent topics across participants while retaining sufficient flexibility to probe individual experiences and meanings [33].

Invitations to participate were distributed to patients with type 2 diabetes through Sweet Home. Participants were eligible if they were over 18 years of age, were in a stable health condition, had been diagnosed with type 2 diabetes for at least six months, consulted healthcare professionals at least twice a year, had participated in the Sweet Home community within the previous month, and agreed to participate in the study.

The interviews were conducted by T.Y., a male associate professor, and H.C., a female researcher, both of whom had experience in health-related research and qualitative studies. Both interviewers had training and experience in qualitative research, including semi-structured interviewing and qualitative data analysis. Neither interviewer had a prior personal or professional relationship with any of the participants, and none of the researchers had any personal, professional, or administrative relationship with Sweet Home. The researchers involved in developing the conceptual framework also participated in data collection and analysis. Reflexive discussions were therefore conducted throughout the research process to critically examine how prior theoretical assumptions might influence data collection and interpretation. Before each interview, participants were informed of the general purpose and procedures of the study and provided informed consent. Throughout the interviews, the interviewers used open-ended questions and follow-up probes to encourage participants to elaborate on their experiences and perspectives. After each interview, the interviewers recorded reflexive notes concerning their assumptions, reactions, and observations. These notes were subsequently discussed within the research team to consider how the researchers’ perspectives might have influenced data collection and interpretation. Before data collection and analysis, the research team recognized that patient-to-patient interactions could involve both supportive and harmful experiences. Therefore, researchers attempted to maintain an open stance toward participants’ diverse accounts rather than assuming that online peer interactions would necessarily enhance future self-continuity.

Eligible patients first completed a brief screening form describing their recent interactions with other patients in the digital community. Two researchers independently reviewed these descriptions and classified each experience according to the six categories identified in Phase 1. Purposive sampling was then used to select two participants whose focal interaction experiences represented each category: beneficial emotional interaction, beneficial informational interaction, beneficial social interaction, harmful emotional interaction, harmful informational interaction, and harmful social interaction. Consideration was also given to variation in age, gender, duration of diagnosis, and treatment status. Sample adequacy was evaluated in terms of information power rather than a numerical saturation threshold. The study had a focused aim, a highly specific sample of patients with type 2 diabetes who were active users of the focal digital community, repeated interviews with the same participants, and a longitudinal within-case and cross-case analytical strategy. Information power was considered in relation to the focused Phase 2 sample as a whole rather than as evidence of saturation within each individual interaction category. Accordingly, two information-rich participants were purposively selected for each of the six interaction categories, yielding 12 participants and 24 interviews across the two interview rounds. The two participants within each category were compared during analysis to examine both convergent and divergent experiences, rather than to establish robust or generalizable differences between the interaction categories.

The first-round interviews explored participants’ illness histories, digital community involvement, experiences of beneficial or harmful interaction, and interpretations of how they related these experiences to the perceived relationship between their present and future selves. Approximately two months later, all 12 participants completed a second-round interview examining whether and how the previously described perceived changes in future self-continuity were reflected in their psychological, existential, and physical quality of life. No participants withdrew between the two interview rounds.

The interview guide was deliberately sequenced so that participants first responded to open-ended questions about their illness experiences, digital community participation, specific interaction experiences, interpretations, and immediate responses before explicit future self-continuity questions were introduced. Similarly, in the second round, participants first reconsidered the earlier interaction and described changes in their views of the future and quality of life before being asked directly about the possible connection among the interaction, their future self, and quality of life. This sequencing provided opportunities for participants to articulate their experiences and future-oriented meanings before the theoretically informed relationship was explicitly probed.

Interviews were conducted electronically through WeChat voice calls. All interviews were conducted by two researchers who had received training in qualitative interviewing and were not involved in the participants’ clinical care. Each interview lasted approximately 60 min. All data collection and initial qualitative analysis were conducted in Chinese. English translation was undertaken only during manuscript preparation and reporting; coding and interpretation were therefore based on the original Chinese-language data. With participants’ permission, interviews were audio-recorded and transcribed verbatim. The interviewers also prepared field notes immediately after each interview to document contextual information, vocal expressions, pauses, emotional tone, initial analytical reflections, and potential issues requiring follow-up. The semi-structured interview guide is presented in Table 2.

Similarly, selected interview quotations were reviewed in their original Chinese context and translated into English by two researchers with overseas study experience, T.Y. and H.C. The researchers carefully considered the original wording, contextual meaning, and participants’ intended meanings when preparing the English versions and cross-checked the translations against the original Chinese quotations. Minor linguistic adjustments were made where necessary to improve readability without altering the substantive meaning.

#### 2.3.2. Data Analysis

The two rounds of interview data were analyzed using hybrid deductive–inductive codebook thematic analysis [34]. Within this hybrid approach, future self-continuity served as a theoretically informed sensitizing construct that guided part of the interview design and analysis, rather than as a theme assumed to have emerged wholly inductively from the interviews. The specific meanings participants attached to their interaction experiences and the interpretive patterns developed from their accounts, however, were not treated as predetermined findings. Data collection and analysis proceeded concurrently, allowing issues identified in earlier interviews to be examined in subsequent interviews. The analysis followed six stages: familiarization with the data, generation of initial codes, development of candidate themes, review of themes, definition and naming of themes, and preparation of the final report [34].

Three coders independently read all transcripts at least twice. During the first-round analysis, the transcripts were initially organized according to the six interaction categories established in Phase 1. Within each category, coding focused on how participants interpreted the interaction, the meanings they assigned to it, and whether and how they associated the interaction with a stronger, weaker, or unchanged perceived connection with their future selves. The categories from Phase 1 provided an initial organizing framework, whereas the higher-order interpretive themes concerning participants’ future selves were developed through analysis of the Phase 2 interview data.

The study was not originally designed to formally compare spontaneous future-oriented references with responses elicited after direct future-self prompts as separate analytic categories. Accordingly, responses to the focused future-self questions were interpreted as theoretically informed accounts of participants’ perceptions rather than as independent evidence of a mediating process. The use of focused, theory-informed interviewing together with iterative qualitative analysis is consistent with prior health-service research [9].

The second-round transcripts were coded for participants’ accounts of psychological, existential, and physical quality of life. A longitudinal case matrix was created for each participant to examine the interaction experience and interpretation described in the first interview alongside the quality-of-life account provided in the second interview. The researchers first conducted within-participant comparisons across the two time points and then compared patterns across participants and interaction categories. These comparisons were used to examine convergent, divergent, and mixed accounts rather than to infer stable differences between the six interaction categories. Particular attention was given to accounts that differed from the dominant patterns.

To distinguish future self-continuity from quality-of-life accounts, excerpts were interpreted according to their primary analytic meaning and contextual focus. Statements describing participants’ perceived connection, continuity, or similarity between their present and future selves were considered related to future self-continuity, whereas statements describing evaluations of their current emotional state, physical condition, meaning in life, or overall well-being were considered quality-of-life accounts. When a participant discussed future-oriented perceptions and current well-being within the same narrative, the coders did not treat the presence of a shared concept such as meaning, purpose, or perceived control as sufficient to assign the excerpt to both constructs. Instead, they considered the primary referent of the statement, its temporal orientation, and the surrounding context to determine whether the excerpt primarily concerned the connection between present and future selves or an evaluation of current well-being. Where the two meanings could be analytically distinguished within a longer passage, the passage was divided into meaning-bearing segments and coded accordingly. Where they could not be meaningfully separated without disrupting the participant’s intended meaning, the excerpt was assigned according to its predominant analytic focus. This approach was used to maintain a clear conceptual distinction between the two constructs while preserving the contextual meaning of participants’ accounts.

The same three coders (J.L., Y.Z., and J.C.) independently reviewed the initial codes and discussed discrepancies until consensus was reached. The coding framework was continuously reviewed and refined throughout the analysis process. The coding unit was each meaning-bearing segment of the interview transcript. Inter-coder agreement was assessed using Holsti’s coefficient of reliability [30]. As in Phase 1, the coefficient for each pair of coders was calculated as CR = 2M/(N_1_ + N_2_), where M represents the number of coding decisions on which the two coders agreed and N_1_ and N_2_ represent the total number of coding decisions made by the respective coders. For the three coders, Holsti coefficients were calculated for each pair of coders and then averaged to obtain an overall agreement coefficient. The coding unit for this calculation was each meaning-bearing segment of the interview transcripts. The resulting mean coefficient was 0.91, exceeding the recommended threshold of 0.80 [30]. An audit trail documenting coding decisions, theme development, analytical memos, and research team discussions was maintained throughout the analysis. Representative quotations were selected to illustrate the identified themes while preserving participants’ intended meanings.

### 2.4. Ethical Considerations

The study was conducted in accordance with the Declaration of Helsinki and was approved by the Institutional Review Board of the authors’ university (Approval No. IRB-BUAA-SEM-2025-0806). Permission to recruit participants was also obtained from the digital healthcare community.

For Phase 1, individual informed consent was waived because only publicly accessible posts were analyzed, in accordance with the ethical approval obtained for this study. To reduce the risk of deductive identification, quotations were translated into English and lightly paraphrased where necessary to reduce searchability while preserving their original meaning; usernames and other potentially identifying details were removed. Before data collection, all participants received written and verbal information concerning the purpose of the study, interview procedures, audio recording, confidentiality, voluntary participation, and their right to withdraw without affecting their treatment. Written informed consent was obtained before the first interview and reconfirmed verbally before the second interview.

Pseudonyms were used in transcripts, tables, and quotations. Names, contact details, and other directly identifying information were stored separately from the interview data. Digital recordings and transcripts were kept in encrypted, password-protected files accessible only to the research team. Participants were informed that anonymized quotations could be included in publications and teaching materials.

### 2.5. Trustworthiness

The trustworthiness of the study was evaluated according to credibility, dependability, confirmability, and transferability [29,35].

Credibility was strengthened through purposive coverage of all six interaction categories, two interviews with each participant, open-ended questioning, follow-up probing, and comparison of accounts across time. At the end of each interview, the interviewers summarized the principal points and invited the participants to clarify or correct the interpretation. Participants were also invited to review brief summaries of the preliminary findings, and no substantive disagreements were reported.

Dependability was supported by using a consistent semi-structured interview guide, documenting recruitment and interviewing procedures, and maintaining an audit trail of transcripts, coding frameworks, analytical decisions, and theme revisions. Regular research-team meetings were held to review the development of codes and themes.

Confirmability was promoted through independent coding by three researchers, consensus discussions, reflexive field notes, and attention to accounts that contradicted or complicated the emerging interpretation. After each interview, the interviewers documented their assumptions, reactions, and reflections on how their perspectives or questioning might have influenced the interview process. These reflexive notes were subsequently considered during research-team discussions to examine and make explicit the potential influence of researcher perspectives on data collection and interpretation.

Transferability was facilitated by providing detailed descriptions of the digital community, eligibility criteria, participant characteristics, interaction categories, interview procedures, and representative quotations. These contextual details enable readers to assess the relevance of the findings to similar digital healthcare communities and chronic illness populations.

## 3. Results

### 3.1. Demographic Characteristics

A total of 12 patients with type 2 diabetes participated in the study and completed both rounds of interviews. The sample included seven women and five men, aged between 27 and 62 years, with a mean age of 44.1 years. The duration since diagnosis ranged from six months to 11 years, with a mean duration of 4.5 years. Participants represented a range of occupational backgrounds, including service work, accounting, education, sales, freelance work, photography, and retirement.

Six participants described predominantly beneficial interaction experiences, while six described predominantly harmful experiences. Within each group, two participants represented emotional interaction, two represented informational interaction, and two represented social interaction. Four participants were categorized as well-controlled/asymptomatic, four were receiving oral medication, three were using insulin, and one was receiving hospital treatment. Participant characteristics are presented in Table 3.

### 3.2. Themes and Sub-Themes

As shown in Table 4, the analysis generated three overarching themes and nine sub-themes. The six emotional, informational, and social interaction sub-themes within the first two overarching themes corresponded to the Phase 1 categories used to organize purposive sampling, whereas the overarching interpretive themes concerning future self-continuity and quality of life were developed from the Phase 2 interview accounts. The first theme described how participants perceived beneficial emotional, informational, and social interactions as strengthening the continuity between their present and future selves (i.e., future self-continuity). The second theme described how participants perceived harmful forms of these interactions as disrupting this continuity. The third theme concerned how participants described perceived changes in future self-continuity in relation to their subsequent accounts of psychological, existential, and physical quality of life.

#### 3.2.1. Beneficial Patient-to-Patient Interactions and Patients’ Future Self-Continuity

Beneficial interactions were described as relevant to more than patients’ immediate emotions, knowledge, or social experiences. Participants interpreted these interactions as evidence that illness could remain manageable, that current actions could shape future health, and that a meaningful future remained possible. Across the three interaction categories, participants perceived beneficial experiences as strengthening future self-continuity by restoring hope, clarifying future pathways, reinforcing perceived control, and creating a sense of shared continuity with others.

##### Beneficial Emotional Interaction

Beneficial emotional interactions involved expressions of understanding, encouragement, empathy, and care. Participants described such exchanges in relation to reduced helplessness and fear following diagnosis. Emotional reassurance was also described as helping participants reinterpret diabetes as a continuing but manageable part of life rather than as the end of their previous identity and future possibilities.

Black Cat initially experienced diagnosis as a sudden rupture between his previous life and the future he had expected. He described encouragement from other patients as helping him regain a sense that his future remained reachable:

*“When I was diagnosed with diabetes, I thought my sky was falling. The patients on the forum became my life and spiritual support. Their care dispelled my helplessness and worry, and their encouragement slowly changed my attitude toward life and my future. Now, communicating with other patients helps me know the direction of my future life and makes me change my unhealthy habits.”* *—Black Cat, male, 43 years*

Health Account similarly described how emotional sharing was associated in her account with less fear of heredity and a stronger sense of being part of a continuing community rather than an isolated patient:

*“When I was diagnosed, I was depressed and felt inferior. Through communicating with friends online, I began to think my situation was not as serious as I had imagined. It feels like a family. Everyone talks openly about happiness and pain. It gives newly diagnosed patients confidence to continue living and hope for their future.”* *—Health Account, female, 27 years*

These accounts suggest that participants perceived beneficial emotional interaction as strengthening future self-continuity by reducing their emotional distance from the future. Participants described becoming more able to imagine themselves continuing to live, adapt, and pursue future goals as fear and helplessness diminished.

##### Beneficial Informational Interaction

Beneficial informational interactions provided patients with practical knowledge, experiential advice, and concrete strategies for disease management. Participants did not perceive such information merely as factual assistance. Rather, they described it as helping them establish a clearer connection between present behavior and future health outcomes.

Angel Light described how advice concerning exercise was related in her account to a shift from an initially hopeless response toward a greater sense of control:

*“At first, I could not accept the diagnosis. I did not even want to be treated. Patients on the forum said that running could improve physical fitness and reduce blood sugar, so I followed their suggestions. After seven months, my weight and blood sugar had decreased. They not only told me how exercise could help but also gave me expectations and hope for the future. I now believe I can control my future health.”* *—Angel Light, female, 38 years*

Hope in Future similarly interpreted the accumulated advice of other patients as a practical route toward a longer and healthier life:

*“Friends gave many useful suggestions, such as maintaining regular sleep and meal times, exercising every morning and evening, and controlling diet scientifically. I believe that if you remain disciplined, positive, and hopeful about the future, you can live a long and healthy life.”* *—Hope in Future, female, 55 years*

These narratives suggest that participants perceived beneficial informational interaction as making the future appear more predictable and actionable. Knowledge obtained from other patients was described as linking present self-management practices to an anticipated future self who remained healthier, more capable, and more independent.

##### Beneficial Social Interaction

Beneficial social interactions enabled patients to form friendships, receive companionship, and develop a sense of belonging. Participants described these relationships as providing continuity between their present illness experience and a future in which they would remain socially connected and valued.

Sugar Controller explained that relationships with other patients were associated in her account with a shift from isolation toward reciprocal commitment:

*“I once had no confidence in my future and felt that I might not live for long. On Sweet Home, I found many patients like me, and several became good friends. They told me that we could travel further on the road to controlling blood sugar together. These friends give me hope to continue living. I also give them support because they need me to encourage their future.”* *—Sugar Controller, female, 54 years*

Eastward River described the community as a “spiritual home”, noting that the friendships and close relationships he developed there were accompanied by a stronger motivation to protect his health:

*“I lived alone and lacked the company of family and close friends. I once lost hope and began to abandon myself. Through the local group on the forum, I met people with similar experiences and later met my girlfriend. Friendship and love made me learn self-discipline and cherish my body. We will face difficulties together and welcome our future.”* *—Eastward River, male, 29 years*

Beneficial social interaction was therefore perceived by participants as strengthening future self-continuity through relational belonging. Participants imagined their future selves not only as surviving patients but also as friends, partners, and valued members of a continuing social network.

#### 3.2.2. Harmful Patient-to-Patient Interactions and Patients’ Future Self-Continuity

Participants described harmful interactions in relation to future selves that appeared threatening, uncertain, or disconnected from their current identities. Emotional distress, conflicting information, interpersonal betrayal, and deceptive relationships were associated in participants’ accounts with weaker confidence in future health and a reduced perceived usefulness of current self-management efforts.

##### Harmful Emotional Interaction

Harmful emotional interaction included expressions of despair, hostility, ridicule, and offensive language. Participants described two related processes: they related emotional distress to exposure to other patients’ fear, while direct personal attacks were associated with a diminished sense of self-worth and concerns about future social acceptance.

Far from Reality described repeatedly reading other newly diagnosed patients’ accounts in relation to intensified fear about his own future:

*“Many new patients talked about unstable blood sugar, weight loss, unfair fate, and disappointment in the future. When I read their experiences, the emotional fluctuation became very strong. Their panic made me fall into panic. I began to feel an inexplicable disappointment and helplessness about my own future.”* *—Far from Reality, male, 38 years*

Flying Dancer experienced direct verbal attacks and related these experiences to doubts about both his social value and his place in the future:

*“Two younger people attacked me with very ugly words and said that I was old and confused. My heart was badly hurt. I began to doubt society and myself. Does society still need me in the future? Can I still be accepted by the people around me? Sometimes I feel that I am becoming older and that there is no value for me in the future.”* *—Flying Dancer, male, 57 years*

Participants perceived these experiences as disrupting future self-continuity and related temporary emotional distress to broader negative evaluations of their future selves. Participants no longer saw the future as an extension of their current lives but as a period characterized by worsening illness, rejection, or diminished personal value.

##### Harmful Informational Interaction

Harmful informational interaction involved disputes, contradictory claims, unverified advice, and catastrophic interpretations of disease. Rather than increasing understanding, participants associated these exchanges with greater uncertainty and reduced confidence in their own judgments.

Candy described repeated arguments as evidence that she might become increasingly unable to manage her emotions:

*“There were many quarrels on the forum, and they always put me in a bad mood. When I encounter different opinions, I cannot stop myself from arguing. I do not know whether this is because of the illness. Can I control my emotions in the future? Will my mood become worse, or will I develop more extreme behaviors?”* *—Candy, female, 49 years*

Cool Heart encountered alarming claims about heredity during pregnancy. She related these messages to greater difficulty imagining a coherent future for herself and her child:

*“Many patients’ words made me feel worse and more depressed. They said that the child might develop diabetes later and continue our pain. I did not know whether the child had hope or whether I had a future. I did not know whether keeping or giving up the baby was the responsible choice. I felt distressed and helpless.”* *—Cool Heart, female, 33 years*

Participants perceived harmful informational interactions as disrupting future self-continuity in relation to greater ambiguity around future health, family roles, and personal decision making. Participants became less confident that their current judgments could lead to a stable or desirable future.

##### Harmful Social Interaction

Harmful social interaction involved the exploitation or betrayal of relationships that patients had initially perceived as supportive. Participants associated deceptive promotion and privacy violations with damaged trust and greater caution about seeking future support.

Dying Slowly developed an apparent friendship with another community member who directed him to a potentially fraudulent healthcare provider:

*“Gradually, we became so-called online friends. I believed his recommendation and spent nearly 30,000 yuan at the hospital. Later, I discovered that the hospital was suspected of providing fraudulent services and that he might have been promoting it. Since then, I have been angry and depressed. I worry that the medicine may damage my body and that my physical condition will become worse. Sometimes I lose confidence in future treatment.”* *—Dying Slowly, male, 62 years*

A Tired Heart described the disclosure of her health information and information about her children in relation to increased uncertainty about her future:

*“I trusted this friend and told her almost everything. Later, I found that she had discussed my blood glucose results and information about my children with other people on the forum. It affected my personal life and my children. Social discrimination already causes me pain, and I do not know whether this incident will negatively affect our future lives.”* *—A Tired Heart, female, 44 years*

These accounts suggest that participants perceived harmful social interaction as disrupting future self-continuity in relation to damage to the relational resources through which they had expected to obtain future support. Participants described trust being replaced by vigilance, regret, and concern about continuing health and social consequences.

#### 3.2.3. Perceived Changes in Future Self-Continuity in Relation to Patients’ Accounts of Quality of Life

The second-round interviews indicated that participants’ perceived changes in future self-continuity were reflected in their accounts of three interconnected dimensions of quality of life. Participants who described stronger future self-continuity generally described greater emotional stability, stronger meaning and control, and more sustained health-management behavior. Participants who described disrupted future self-continuity reported persistent fear, diminished meaning, and worsening perceptions of their physical condition and functional capacity.

##### Psychological Quality of Life

Participants who described stronger future self-continuity also reported greater hope, confidence, and emotional stability. They described how imagining a manageable future was related to their ability to reinterpret temporary fluctuations in blood sugar or mood without viewing them as evidence of inevitable decline.

*“I still worry when my blood sugar changes, but I no longer think that everything is over. I can picture myself living normally several years from now, so I calm down more quickly and do what I need to do today.”* *—Black Cat, second-round interview*

By contrast, disrupted future self-continuity was associated with recurring anxiety and anticipatory fear. Participants continued to interpret other patients’ negative experiences as possible descriptions of their own future:

*“Even when my results were stable, I kept thinking about the people who said they were getting worse. I felt that their future might become my future. This made me nervous and sometimes unable to sleep.”* *—Far from Reality, second-round interview*

The findings suggest that psychological quality of life was associated, in participants’ accounts, with whether they could envision a future self who remained recognizable, manageable, and worthy of present investment.

##### Existential Quality of Life

Participants who described a stronger connection with their future selves also reported greater meaning, purpose, and direction in their current lives. Self-management was interpreted not merely as compliance with medical advice but as an investment in the person they intended to become.

*“I now have a clearer goal. I want to remain independent and continue taking care of my family. Controlling my diet and exercising are not only about today’s blood sugar; they are about the kind of life I want to have later.”* *—Hope in Future, second-round interview*

Conversely, participants who described disrupted future self-continuity reported reduced personal value and uncertainty regarding whether future effort remained meaningful:

*“After being attacked, I sometimes thought that perhaps an older patient had nothing useful to offer. When I could not see any value in my future self, I also lost interest in planning what I wanted to do next.”* *—Flying Dancer, second-round interview*

Thus, existential quality of life was reflected in whether patients perceived their present efforts, identities, and relationships as continuing into a meaningful future.

##### Physical Quality of Life

Participants who described stronger future self-continuity also perceived connections between their future-oriented self-perceptions and exercise, diet, medication adherence, sleep, energy, and everyday functioning. They described a valued future self as giving current health behavior a clearer purpose.

*“I continued running because I could see the person I wanted to become. My sleep and energy improved, and I was able to work more normally. When I think about my future self, it becomes easier to continue even when exercise is tiring.”* *—Angel Light, second-round interview*

Participants who described disrupted future self-continuity more frequently described fatigue, poor sleep, heightened attention to bodily discomfort, and uncertainty regarding treatment:

*“After the false treatment experience, I became suspicious of almost every suggestion. I worried that my body had already been damaged. I slept poorly, felt tired during the day, and sometimes delayed making decisions about treatment because I no longer knew whom to trust.”* *—Dying Slowly, second-round interview*

These patterns were not uniform across all participants. Some participants described no noticeable change or only temporary effects, whereas others attributed changes in their well-being primarily to clinical treatment, disease progression, or personal life circumstances rather than to the online interaction. These divergent accounts were retained in the analysis and indicate that the interpretive sequence described above was neither universal nor deterministic.

Overall, the longitudinal accounts indicate an interpretive sequence in which patient-to-patient interaction experiences were associated with how participants understood their future selves, and these future-oriented interpretations were subsequently reflected in their accounts of emotional states, sense of meaning and control, and perceived physical functioning.

## 4. Discussion

### 4.1. Theoretical Implications

This sequential qualitative study identified 25 beneficial and harmful patient-to-patient interaction practices across emotional, informational, and social domains in a digital healthcare community. The interview findings further suggest that patients interpreted these interactions in relation to their future selves: supportive exchanges were associated with hope, perceived control, and belonging, whereas harmful exchanges were associated with fear, uncertainty, and mistrust. These future-oriented interpretations were subsequently described in relation to participants’ psychological, existential, and physical quality of life. Taken together, the findings provide a more differentiated understanding of online patient exchange as a process that may simultaneously facilitate empowerment and expose patients to emotional, informational, and relational risks [1,2,3,5].

First, this study develops an integrated framework for the dual nature of patient-to-patient interaction. Whereas prior research has emphasized social support, knowledge exchange, and empowerment [1,2,9,25,26], harmful experiences have typically been examined separately as misinformation, privacy risks, or interpersonal conflict [3,27]. The findings show that beneficial and harmful practices coexist within emotional, informational, and social interactions: each domain may either create support and value or generate distress, confusion, and relational harm. Their perceived implications therefore depend on the specific practices involved and how patients interpret them, extending research from whether interaction occurs to how patients experience and interpret value-creating or value-destroying outcomes [5,23,24,31]. Importantly, the negative valence of an interaction should not be equated with patient-safety risk, because difficult illness narratives or legitimate disagreement may sometimes provide realistic or corrective information and therefore should not be considered harmful solely on the basis of their negative or conflicting nature. More broadly, the findings point to important distinctions among unsupported experiential advice, misinformation, disinformation, and commercially motivated deception: these forms of content may differ in their evidentiary basis, intentionality, and potential implications for patient safety.

Second, the findings extend future self-continuity research by highlighting its interactional and context-dependent nature. Chronic illness may disrupt established identities, life plans, and anticipated futures [8]. In this study, participants described beneficial emotional interaction in relation to reduced fear and a view of illness as manageable rather than as an irreversible rupture. They related practical knowledge to a clearer connection between present self-management behaviors and anticipated future health, while companionship and belonging were described in relation to imagining their future selves as socially connected and valued. Future self-continuity was therefore described not only as an internal temporal cognition but also in relation to the emotional, informational, and relational resources encountered through patient-to-patient interaction. This finding broadens research that has primarily examined future self-continuity in relation to individual decision making and health behavior [6,7].

Importantly, these interactional processes should be interpreted within the specific sociocultural context in which this study was conducted. In China, digital patient communities may function not only as channels for health information exchange but also as spaces for interpersonal trust building, emotional support, and collective coping with chronic illness. Cultural expectations regarding family responsibility, social belonging, and reliance on trusted relationships may influence how patients evaluate peer experiences and incorporate them into their understandings of future health. Meanwhile, the authority traditionally associated with healthcare professionals may shape how patients negotiate between professional recommendations and experiential knowledge shared by peers. Therefore, the role of patient-to-patient interaction in supporting or disrupting future self-continuity may partly depend on cultural and healthcare contexts rather than representing a universally identical process across all digital health communities.

Third, this study provides a temporal and interpretive account of how participants related patient-to-patient interaction to quality of life. Previous studies have established associations between online social support and patients’ quality of life [9,24,25]. Recent healthcare research has likewise emphasized that improving health-related quality of life requires attention to psychological and social needs alongside clinical management [36]. The present findings extend this literature by illustrating how participants incorporated interaction experiences into their understandings of their future selves. When patients could imagine a future self that remained recognizable, manageable, and personally valuable, they described greater hope, emotional stability, meaning, perceived control, and commitment to self-management. When the future self appeared threatening, valueless, or uncontrollable, they described anxiety, reduced meaning, sleep difficulties, bodily concerns, and uncertainty about treatment. These patterns are consistent with evidence linking future self-continuity to health behavior, depression and hopelessness, and meaning in life [7,13,14]. Importantly, these accounts represent participants’ perceived connections between future self-continuity and their physical well-being rather than evidence that changes in physical functioning were caused by future self-continuity. However, these patterns were not consistent across all participants. Some reported no noticeable change or only temporary effects, while others attributed changes in well-being primarily to clinical treatment, disease progression, or personal life circumstances rather than online interaction.

### 4.2. Practical Implications

The findings have implications for the governance of digital healthcare communities and the provision of patient support. Participants’ accounts directly highlighted the importance of constructive communication, ongoing support, and protection from harmful interactions. Building on these participant-grounded findings and relevant previous research, platform administrators could assess the quality of emotional, informational, and social exchanges rather than relying solely on membership, posting frequency, or interaction volume [37]. This suggestion represents a researcher-derived design implication rather than a governance practice directly tested in the present study.

Community guidelines and human moderation could be used to address hostile language, repeated interpersonal conflict, privacy violations, and deceptive commercial activity. At the same time, governance responses should be proportionate to the nature and potential risk of the content rather than triggered simply by negativity or disagreement. Unsupported experiential advice may warrant clearer source attribution and explicit differentiation from professional medical advice, together with opportunities for professional verification. Potentially harmful misinformation involving medication, heredity, pregnancy, treatment modification, or other clinically consequential issues may require risk warnings or professional review. Where available evidence indicates deliberate deception or undisclosed commercial interests, more targeted moderation and clearer commercial disclosure may be warranted. Legitimate disagreement and accounts of difficult illness experiences, by contrast, should not be automatically suppressed merely because they are negative or conflicting, provided that they do not present unsupported claims as established medical guidance or otherwise create a substantial patient-safety risk. Automated monitoring may also assist in identifying potentially harmful content, but this should be regarded as a potential governance option rather than an established solution, and its use should account for false positives, contextual misclassification, privacy concerns, and the risk of suppressing legitimate expressions of distress. Posts expressing severe despair, anxiety, or treatment abandonment may require timely supportive responses and referral to professional services. Stronger privacy safeguards and clearer identification of commercial content may also be warranted, particularly when promoters present themselves as fellow patients to gain trust [2,3,27].

Healthcare professionals should discuss patients’ use of digital communities as part of routine care. Patients may benefit from guidance in distinguishing individual peer experiences from general health information and personalized clinical advice. Professionals can also help patients translate credible online information into realistic future-oriented health goals. Connecting present self-management practices with an attainable and meaningful future self may help support patients’ future self-continuity as a psychological resource relevant to long-term well-being [7]. These suggestions should be understood as practice-oriented implications derived from the study’s findings and informed by prior literature, rather than as interventions whose effectiveness was directly established by the present qualitative evidence. This recommendation is also consistent with broader developments in AI-enabled healthcare, which emphasize coordination across healthcare institutions and relevant stakeholders and highlight the importance of governance in the responsible implementation of emerging medical technologies [38].

### 4.3. Limitations

Several limitations should be acknowledged. First, the study was conducted within a single Chinese digital diabetes community. The findings should therefore be interpreted within this specific sociocultural and healthcare context, where context-specific expectations regarding family responsibility, interpersonal trust, social belonging, healthcare authority, and peer advice may shape how patients engage in patient-to-patient interaction and construct future self-continuity. Accordingly, the findings should not be assumed to transfer uniformly to other countries, healthcare systems, digital platforms, or chronic conditions. Second, Phase 2 recruitment through Sweet Home may have introduced selection bias related to continued community engagement. Patients who disengaged from the community because of harmful experiences, passive users or lurkers, and individuals with lower digital literacy may have been less likely to participate. This issue is particularly relevant because the study examined both beneficial and harmful patient-to-patient interactions; individuals with more negative experiences may have been less available for recruitment. Future research should consider recruiting beyond active digital community members and incorporating perspectives from inactive users, former users, or patients with varying levels of digital engagement. Third, Phase 2 participants were purposively sampled across the six interaction categories identified in Phase 1. Although these categories guided sampling and the initial organization of the interview material rather than predetermining the higher-order interpretive themes, this strategy may have limited the representation of mixed or overlapping interaction experiences. With only two participants associated with each focal interaction category, the study was not designed to establish saturation within individual categories or robust differences among emotional, informational, and social interaction processes. In addition, organizing recruitment around a focal interaction experience may have reduced the visibility of ambivalent, mixed-valence, or transitional experiences, even though participants were able to describe experiences beyond their sampling category. Future research could examine more directly how supportive and harmful interactions coexist, change over time, and may be interpreted differently by different patients. Fourth, the Phase 2 interview guide explicitly explored participants’ perceptions of future self-continuity and quality of life, consistent with the study objectives. This theory-informed approach may nevertheless have introduced a degree of prompting or confirmation bias, as participants may have been encouraged to interpret their experiences in relation to these constructs even when such connections were not initially salient to them. Although the interviews remained semi-structured and included open-ended questions, follow-up probing, and attention to divergent or contradictory accounts, the explicit focus on future self-continuity and quality of life may have influenced both data generation and subsequent interpretation. The resulting findings should therefore be understood as participants’ interpretations elicited within a theoretically informed qualitative design rather than as independent evidence of a mediating mechanism. Future research could incorporate more open-ended interviews that initially explore participants’ experiences without explicitly foregrounding the proposed relationship between future self-continuity and quality of life, which would allow a clearer distinction between spontaneously emerging future-oriented meanings and interpretations elicited through direct theoretical prompting. Fifth, the two-month interval captured relatively short-term perceived changes and could not reflect longer-term fluctuations in future self-continuity or quality of life. Changes described by participants may also have reflected disease progression, clinical treatment, family circumstances, employment, or other life events. Longer qualitative longitudinal studies with additional interview waves would provide a fuller account of how these interpretations develop, stabilize, or change [20,21]. Finally, the study relied on participants’ subjective accounts and did not establish causal relationships among patient-to-patient interaction, future self-continuity, and quality of life. Future research could combine multi-platform data, longitudinal behavioral records, interviews with healthcare professionals, and quantitative or experimental methods to further examine the proposed interpretive process.

## 5. Conclusions

This study suggests that patient-to-patient interaction in digital healthcare communities may be experienced as both supportive and harmful. Emotional, informational, and social interactions may each be experienced as supportive or harmful, and participants interpreted these experiences in relation to the continuity between their present and future selves. Our findings further suggest that future self-continuity provides a useful interpretive perspective for understanding how patients connect their online interaction experiences with different aspects of quality of life. Rather than viewing digital healthcare communities as inherently beneficial, platform designers and healthcare professionals may benefit from fostering supportive interactions while addressing misinformation, harmful interpersonal conflict, privacy risks, and deceptive promotion. By integrating future self-continuity into research on digital healthcare communities, this study extends current understanding of how patients perceive and make sense of online interactions in relation to their well-being and offers practical insights for developing safer and more patient-centered digital health services. However, these findings should be understood within the specific sociocultural and healthcare context of a Chinese digital diabetes community. Further longitudinal research across different populations, platforms, and healthcare settings is needed to examine how these interpretations develop over time.

## Figures and Tables

**Table 1:** Summary of coding scheme of patient-to-patient interaction in digital healthcare community (Sweet Home).

Effect	Category	Definition	Activity Theme	Representative Quote	Proportion (%)
Beneficial	Emotional interaction	Share, express, and/or sense the positive, valuable, or helpful feelings, mood, or mental images on the forum.	Compliment people or things	“Look at your photos. I find you are much thinner and healthier. How I envy you!”	21.2
			Understand peers	“Facing delicious food. But you can’t open your mouth. I understand your pain.”	
			Sympathize with peers	“Xiao Zhang has been in hospital for half a month. She’s in serious condition. It’s not easy for a single girl like her to live in a big city.”	
			Encourage peers	“I think your blood sugar has been controlled very well. Keep going and keep exercising.”	
			Care about peers	“You need to take care of yourself. I look forward to seeing you again when you come back from the hospital.”	
			Bless peers	“Congratulations on finding your lover. Our forum is almost becoming a marriage website. Haha.”	
	Informational interaction	Argue, review, and/or comment positively or constructively on the complexity and subtleties of people and things on the forum.	Exchange know-how or answers to questions	“Comparison of the results of the blood glucose meter by Roche, Johnson, and Bayer. Continuous updating!”	29.2
			Share practical skills, experiences, and/or expertise on dealing with disease	“Diabetes can be prevented. Pay attention to the following ten points, which are conducive to the prevention of complications.”	
			Explain and/or teach how to use a medical service	“I bought a new type of blood glucose meter with a USB interface used to download data. If you don’t know how to use it, feel free to contact me.”	
			Engage in interactive learning with each other	“Dr. Liu makes a series of teaching videos. Update at 8:00 pm every night. Let’s join his micro-blog to learn together.”	
			Confirm or approve peers’ ideas, comments, suggestions, and advice based on one’s own experience	“What you said about quitting smoking works. I’ve been doing it for half a year, and now I smoke less. Other smokers might try it.”	
	Social interaction	Bond, form, expand, and/or foster the interpersonal or collective relationships and camaraderie with peers on the forum.	Develop or find friends	“I am in Jining, Shandong Province. Are there any friends from the same city to communicate with me?”	19.4
			Gain companionship through verbal communications with peers	“Let’s talk with our sugar friends about the plan to travel on national day …. I feel much better.”	
			Build a professional or regional group for healthcare or friendship	“Are there any Sichuan friends in the group? How about having a party at the weekend?”	
			Establish a sense of relatedness or belonging to an organization	“We have nearly 100 friends in this Guangdong local group. Welcome new friends!”	
Harmful	Emotional interaction	Express and/or sense the negative, unhelpful, or harmful feelings, mood, or mental images on the forum.	Express disappointment, helplessness, or even destructive words to peers	“Getting sick is God’s punishment for us. Should we continue to live?”	9.5
			Post content with hostile or rude words	“Don’t talk nonsense here. You’re so stupid. You’re not only sick physically, but also mentally. Get out!”	
	Informational interaction	Argue, review, and/or comment negatively or destructively on the complexity and subtleties of people and things on the forum.	Quarrel with peers over the uncertainty of symptoms, therapeutic regimen, or even meaningless issues	“A: Judging from your test sheet, your blood sugar is not high. Keep exercising is OK. B: Exercise? Then why do you need medicine? Do you really understand? Don’t pretend to understand. A: Can you understand better than I do?”	11.6
			Offer unsolicited or receive poor suggestions and advice	“I have heard some old Chinese doctors say that eating frogs can cure diabetes. Do you want to give it a try?”	
			Complain about a medical service or other people, such as poor curative effect or peers’ comments	“You always said exercise was good. I don’t believe that exercise can cure diabetes at all. Don’t lie to me.”	
			Slander other peers or things	“A: Never use the ABC brand insulin pump; the quality is too poor! B: Are you sure? I’m fine with it. Are you the shill of another brand?”	
			Belittle peers	“Don’t believe what he says. He has only been at the forum for a few days.”	
			Ask the same questions many times, but no response from peers	“Does anyone know what my condition is? No one’s answered yet?”	
	Social interaction	Damage interpersonal or collective relationships with peers or take advantage of the pseudo-camaraderie with peers to carry out harmful activities.	Disclose or discuss others’ private information	“A friend of mine, XYZ. She works in the same company as you. She has had diabetes for at least five or six years. Her mother is also diabetic. You can ask her.”	9.1
			Sell or promote unidentified or even fake medical products/services to peers	“Have you found the guy who sold the fake blood glucose meter on the forum? I was cheated by him.”	

Note. Percentages represent the proportion of coded discussion topics within the analyzed corpus. Although multiple interaction practices could be identified within a discussion thread during practice-level coding, each valid discussion topic was assigned to one predominant interaction category for the six-category classification reported in this table. Each percentage was calculated as the number of discussion topics assigned to the corresponding category divided by the total number of valid discussion topics (n = 871) and multiplied by 100. The six category-level proportions were therefore mutually exclusive and summed to 100%. These percentages describe the distribution of interaction categories within the analyzed corpus and should not be interpreted as estimates of the prevalence of these interaction practices among all Sweet Home users.

**Table 2:** Semi-structured interview guide for Phase 2.

Interview Round	Research Area	Guiding Questions
First round	Opening and illness background	Please describe your experience of living with type 2 diabetes. What aspects of the illness or its management are currently most important in your daily life?
	Digital community participation	Please describe how you use the Sweet Home community. What usually brings you to the community? What kinds of exchanges do you have with other patients?
	Specific interaction experience	Please think of a recent interaction with another patient in the community that was particularly memorable or meaningful to you. What happened? What did the other patient say or do? How did you respond?
	Interpretation of the interaction	How did you understand the other patient’s words, actions, or intentions? What made you interpret the interaction in this way? Did your understanding of the interaction change afterward?
	Immediate responses	What thoughts, feelings, or actions, if any, followed this interaction? Did it affect the way you viewed yourself, your illness, your treatment, or your relationships with others? It is also possible that the interaction had little or no effect.
	Future self-continuity (connection between present and future selves)	1. Please imagine yourself approximately one year from now, particularly in relation to living with and managing type 2 diabetes. How do you currently view that future self? 2. How much do you care about the health and well-being of your future self? How positively do you feel toward the person you expect to become? 3. In what ways do you expect your future self to be similar to or different from your present self? 4. How connected to or distant from your future self do you currently feel? Did the interaction discussed earlier make you feel more connected to, less connected to, or unchanged in relation to your future self? Please explain.
	Other relevant influences	Apart from this interaction, what other experiences influenced the way you viewed your future at that time? For example, were your views also affected by symptoms, treatment, family members, healthcare professionals, or other life events? How important was the online interaction compared with these factors?
Second round	Reconsideration of the interaction	During our previous interview, you described a particular interaction with another patient. Looking back, how do you now understand that experience? Has its meaning or importance changed for you?
	Changes in views of the future	Since the first interview, has anything changed in the way you view your future health, future life, or future self? It is also possible that no change has occurred. What do you think contributed to any change or lack of change?
	Overall quality of life	Since the first interview, have you noticed any changes in your well-being or daily life? Please describe the changes that have been most important to you.
	Psychological quality of life	Since the first interview, how would you describe your overall emotional and psychological state? Have you experienced any changes in feelings of depression, nervousness, worry, sadness, or fear of the future? 3. Have these feelings become stronger, weaker, or remained unchanged? In what situations have these changes been most noticeable? 4. What do you think contributed to these changes or to the absence of change?
	Existential quality of life	1. Since the first interview, how would you describe your overall sense of meaning, purpose, and direction in life? 2. Do you currently feel more or less able to pursue and achieve your life goals? 3. Has your sense of control over your life changed? Has the way you value yourself as a person changed? 4. How did these changes, if any, develop, and what do you think contributed to them?
	Physical quality of life	1. Since the first interview, how would you describe your overall physical condition and physical well-being? 2. Have you noticed any changes in pain, fatigue, appetite, or other physical symptoms? 3. Have these physical changes affected your daily activities or your ability to manage your illness? 4. What do you think contributed to these changes or to the absence of change?
	Interpretive connection	Do you see any connection between the interaction discussed in the first interview, the way you currently view your future self, and the changes you have described in your quality of life? Why or why not?
	Alternative and mixed explanations	Were these changes influenced by other factors, such as disease progression, treatment, family circumstances, work, or healthcare experiences? Was the influence of the online interaction lasting, temporary, mixed, or absent?

**Table 3:** Characteristics of participants in interviews.

Nickname	Gender	Age (Years)	Duration of Diagnosis (Years)	Occupation	Types of Patient-to-Patient Interaction	Effect of Patient-to-Patient Interaction	Health Conditions
Black cat	M	43	4	Hotel attendant	EI	Beneficial	In treatment, taking drugs
Health account	F	27	2	Accountant	EI	Beneficial	Well-controlled/Asymptomatic
Angel light	F	38	3	Tally clerk	II	Beneficial	Well-controlled/Asymptomatic
Hope in future	F	55	7	Cleaner	II	Beneficial	Well-controlled/Asymptomatic
Sugar controller	F	54	9	Teacher	SI	Beneficial	In treatment, injecting insulin
Eastward river	M	29	3	Photographer	SI	Beneficial	Well-controlled/Asymptomatic
Far from reality	M	38	0.5	Salesman	EI	Harmful	In treatment, taking drugs
Flying dancer	M	57	3	Cashier	EI	Harmful	In treatment, taking drugs
Candy	F	49	5	Clerk	II	Harmful	In treatment, injecting insulin
Cool heart	F	33	1	Freelance	II	Harmful	In treatment, being in hospital
Dying slowly	M	62	11	Retired	SI	Harmful	In treatment, injecting insulin
A tired heart	F	44	6	Full-time mom	SI	Harmful	In treatment, taking drugs

Note. EI = Emotional Interaction; II = Informational Interaction; SI = Social Interaction.

**Table 4:** Themes and sub-themes identified from the two rounds of interviews.

Theme	Sub-Themes	Interview Round
Beneficial patient-to-patient interactions and patients’ future self-continuity	Beneficial emotional interaction	First round
	Beneficial informational interaction	
	Beneficial social interaction	
Harmful patient-to-patient interactions and patients’ future self-continuity	Harmful emotional interaction	
	Harmful informational interaction	
	Harmful social interaction	
Perceived changes in future self-continuity in relation to patients’ accounts quality of life	Psychological quality of life	Second round
	Existential quality of life	
	Physical quality of life	

## Data Availability

The anonymized transcripts used and/or analyzed during the current study are not available due to confidentiality agreements with participants; however, a minimum dataset may be accessed from the corresponding author on reasonable request.
